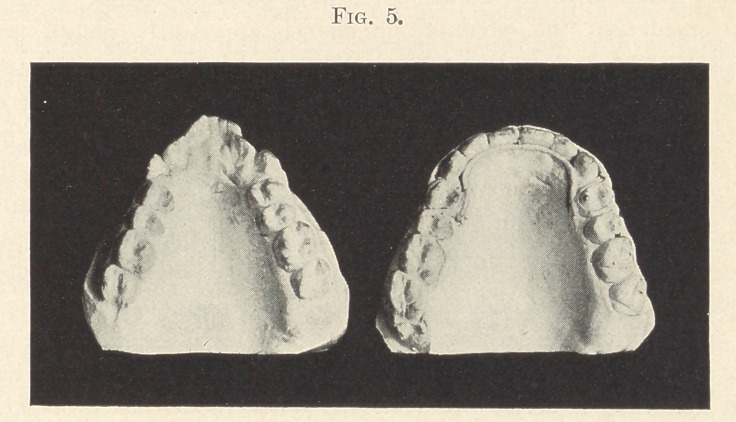# A Report of an Unusual Case of Orthodontia

**Published:** 1905-08

**Authors:** Lawrence W. Baker

**Affiliations:** Boston, Mass.


### A REPORT OF AN UNUSUAL CASE OF ORTHODONTIA.[1]

BY LAWRENCE W. BAKER, D.M.D., BOSTON, MASS.

I SHALL attempt to interest you, gentlemen, for a short time this evening by showing you a series of lantern slides illustrating what, to me at least, is a very interesting case of orthodontia.

This slide (Fig. 1) shows the two side views of the casts before treatment. No doubt your eye falls at once upon the marked deformity in the relation of the two jaws, which at first glance might be considered due to a protrusion of the upper arch, but on consulting the occlusal landmarks it is found that in reality the deformity is caused largely by a decided recession of the lower jaw. Normally, we know, the point of the lower cuspid occupies the space between the upper cuspid and lateral, and in an ordinary case of "distal occlusion" this point falls between the first bicuspid and cuspid, while in this extreme case this same point occupies the space one plane back of the ordinary distal position, or between the two bicuspids. So in simple distal occlusion the jaw recedes one

---

[1] Read before the American Academy of Dental Science, April 5, 1905.

occlusal notch, while in this case the lower jaw recedes not one, but two occlusal notches, to which deformity it seems logical to apply the term *double distal.*

FIG. 1.

That this malformation caused a great impairment of the function of mastication, as well as a pronounced facial disfigurement, can be judged by studying this illustration. I should have hesitated at the responsibility of treating such a case had I not had great confidence in the possibilities of the "Baker anchorage."

FIG. 2.

By way of history, I should like to state that when the child came to me the lower sixth-year molars were beyond saving, so at the proper time the bits of roots were removed, allowing the twelfth-year molars to come forward into the places of the missing sixthyear molars, with the result that you see here. The loss of these natural abutments of the arch greatly complicated the treatment of the case.

Fig. 2 shows another view of the deformity. The effects of the extreme distal position of the lower jaw upon its function is here clearly shown,—the ten or twelve anterior teeth were entirely useless for masticating purposes. It was possible to pass the forefinger between the two arches, even though they were in close apposition, which illustrates their lack of function. This disuse is further indicated by the presence of the cusplets of development, which by use soon wear away.

Fig. 3.

Next (Fig. 3) we have the two side views after treatment. It is clearly seen that the lower jaw has been brought forward to its intended position,—one notch to simple distal and another notch to normal,—greatly improving the facial contour as well as increasing the utility of the entire dental apparatus.

In this case many would have considered extracting in the upper arch necessary; probably the removal of two biscuspids would have been resorted to, or possibly the entire bicuspid region would have been sacrificed. In other words, the upper arch would have been mutilated to correspond with the misplaced lower arch; that is, another deformity would have been created equal to the already existing one. I believe that by keeping the upper arch intact and bringing the receding jaw forward not only were the jaws given full power, but the facial lines were placed in much better balance. Instead of weakening the lower part of the face by moulding it to the weak receding lower jaw, the whole face was strengthened by bringing the chin forward to harmonize with the general facial contour.

In Fig. 4 we see a comparative view of the case. This further illustrates that the jaws and teeth have been brought to their

Fig. 4.

maximum usefulness. This great increase of function is the very secret of the lower jaw remaining in its new position. It was necessary to hold it in its forward position only until the patient unconsciously learned this fact, or until the position became habitual.

This occlusal balance also acts as a perfect natural retainer, the lower arch serving as a form upon which the upper one was moulded, so that later the retainer can be removed without fear of a recurrence of the deformity.

Next we see (Fig. 5) the palatal aspect of the upper arch before and after treatment. Note the great amount of expansion. This expansion was necessary for the proper interlocking of the cusps, for when the lower jaw was brought forward it required a much larger upper jaw than before. The former contracted state was nature's way of compensating for the malposition of the lower arch. The normal placing of this arch required a normal modelling of the upper one. Fig. A shows the contracted condition and B the expanded arch, the difference being indicated by the measurement.

By studying this view it is seen how the lower jaw is locked in its new position. The tendency for it to slip back, even one occlusal notch, to the simple distal position has been prevented, for the utility would be destroyed, there would be no interlocking of the bicuspids and molars, and the cuspids and incisors would have absolutely no antagonism. That the occlusal balance serves as a natural retainer is again clearly shown. The anterior teeth are

Fig. 5.

mechanically retained by means of the device indicated in Fig. B. It will be noted that the teeth posterior to the first bicuspid are not included in the retainer; their new position is maintained by the force of occlusion alone. The bringing forward of this arch just compensated for the expansion of the upper, thus a true occlusal balance is established.

Those of you who have followed this description at all carefully must realize that this case is a typical illustration of the carrying out of one of nature's simple laws, a law that is the very foundation of the science of orthodontia,—the law of occlusion: occlusion for utility, occlusion for retention, and occlusion for facial balance.

## Figures and Tables

**Fig. 1. f1:**
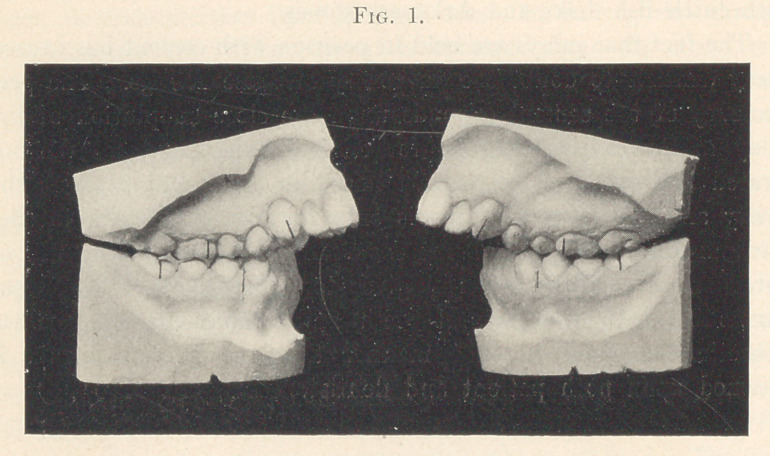


**Fig. 2. f2:**
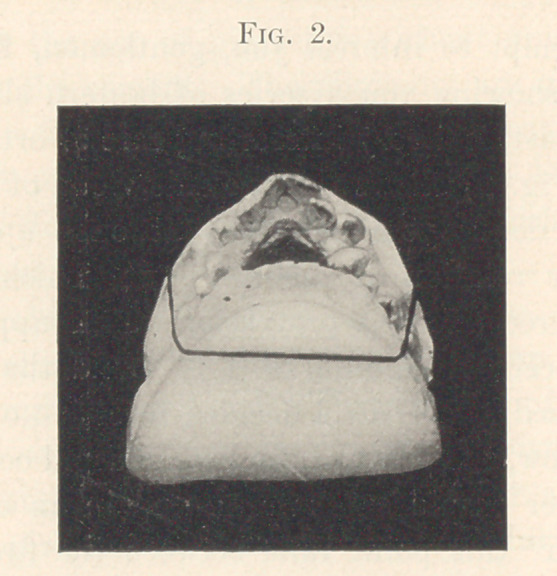


**Fig. 3. f3:**
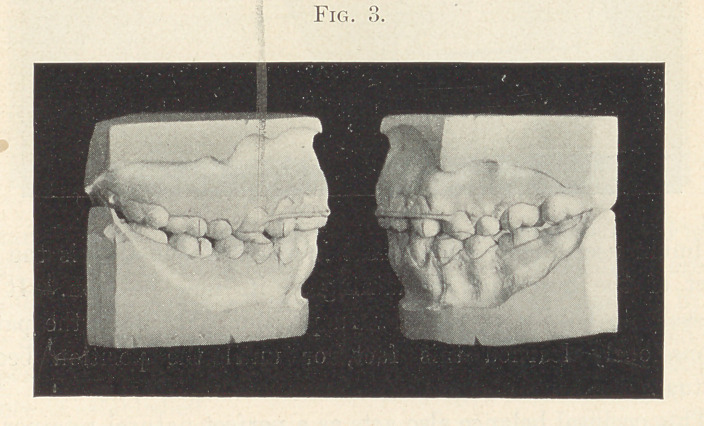


**Fig. 4. f4:**
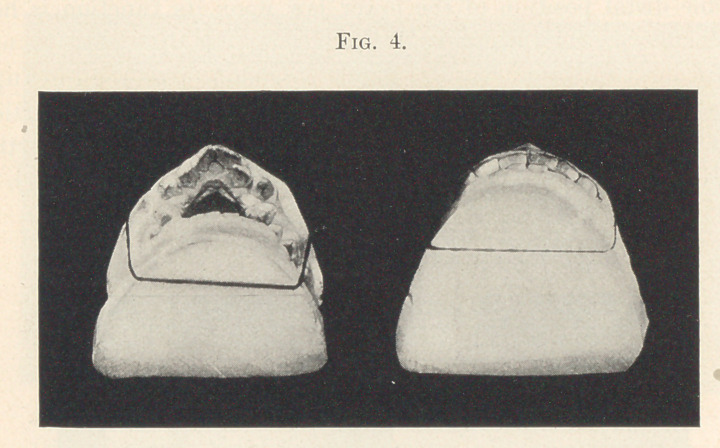


**Fig. 5. f5:**